# Selected Physicochemical Properties of Saliva in Menopausal Women—A Pilot Study

**DOI:** 10.3390/ijerph17072604

**Published:** 2020-04-10

**Authors:** Aleksandra Cydejko, Aida Kusiak, Magdalena Emilia Grzybowska, Barbara Kochańska, Jolanta Ochocińska, Adrian Maj, Dariusz Świetlik

**Affiliations:** 1 Department of Periodontology and Oral Mucosa Diseases, Medical University of Gdansk, Orzeszkowej 18 St, 80-204 Gdansk, Poland; aleksandra.cydejko@gumed.edu.pl (A.C.); adrianm@gumed.edu.pl (A.M.); 2 Department of Gynecology, Gynecological Oncology and Gynecological Endocrinology, Medical University of Gdansk, 80-214 Gdansk, Poland; magdalena.grzybowska@gumed.edu.pl; 3 Department of Conservative Dentistry, Medical University of Gdansk, 80-208 Gdansk, Poland; bkochan@gumed.edu.pl (B.K.); jolao@gumed.edu.pl (J.O.); 4 Department of Biostatistics and Neural Networks, Medical University of Gdansk, 80-211 Gdansk, Poland; dswietlik@gumed.edu.pl

**Keywords:** menopausal women, saliva, ionized calcium, physicochemical properties of saliva

## Abstract

The aim of this study was to estimate differences in selected physicochemical properties of saliva between menopausal and premenopausal women. Methods: The study population consisted of 9 menopausal women and 15 women of the control group. Laboratory tests included the determination of pH saliva, salivary flow rate, and concentrations of lactoferrin, lysozyme, immunoglobulin A, and ionized calcium. Results: Among menopausal women, statistically significant differences were observed in values of salivary flow rate and lysozyme and ionized calcium concentrations; however, no statistically significant differences for pH and concentrations of lactoferrin and immunoglobulin A were found. The salivary flow rate in the study group was significantly lower compared to that in premenopausal women. In relation to lysozyme, statistically significant differences were found between control group and menopausal women to the disadvantage of the latter. However, the concentration of ionized calcium in the saliva of menopausal women was distinctly higher than in the saliva of the control group. Conclusion: The saliva of menopausal women appeared significantly different from that of the control group. Differences in physicochemical parameters such as salivary flow rate and lysozyme and ionized calcium concentrations were observed. These differences in saliva properties observed in menopause can potentially affect the oral environment of women in this particular period, possibly increasing the risk of some pathological changes in the oral cavity and consequently indicating the need to take special care of this group of female patients in order to help them maintain proper oral health. Dentists and gynecologists should be aware of the problems associated with menopause and need to provide these women complete health care, including dental care as an integral part.

## 1. Introduction

Nowadays, dentists, in response to the gradually increasing awareness of the need to maintain oral health, take care of an increasing number of patients in different periods of life. Among them, women in menopausal age are an important group with specific medical conditions and needs. The undesirable symptoms reported by patients during this period are the result of a number of systemic processes occurring in the woman’s body as a consequence of the decrease in estrogenic hormone concentration due to discontinued endocrinological activity of the ovaries [1,2,3,4].

Although the general symptoms that occur in women during menopause are well documented, the level of awareness of the fact that oral discomfort in patients at this time is also an element of menopause complaints is still low. In the female body, deepening biological and hormonal changes are associated with emerging estrogen hormone deficiency. To the best of our knowledge, the oral mucosa, like the vaginal mucosa, contains estrogen receptors. They also occur in the salivary glands [2,5,6,7,8]. Therefore changes in estrogenic hormone levels may have a direct impact on the oral cavity in terms of effects within both the oral mucosa itself and its microflora, as well as the amount of saliva secreted by the salivary glands, and as a consequence may adversely affect teeth and periodontal tissues, resulting in an increased risk of caries and periodontal disease. Estrogen deficiency also affects the maturation process of the oral mucosal epithelium and can lead to its thinning and atrophy, making it more susceptible to local mechanical injuries and causing a change in pain tolerance and problems in the use of removable prosthetic restorations [2,3,4,5,6]. Because of atrophic changes in the oral mucosa, menopausal women can often develop a series of persistent diseases, like burning mouth syndrome, lichen Wilsoni, idiopathic neuropathy, and increased tendency to develop candidiasis due to increased colonization of microorganisms in patients with reduced salivation [4]. Moreover, during the menopausal period, not only the amount of secreted saliva, but also its compositionmay change [5,9]. Individual elements occurring in saliva play a strictly defined role in the proper functioning of the whole organism, nourishing and protecting the surrounding tissues. Moreover, many elements presenting antimicrobial activity are present in saliva, such as immunoglobulins A, lysozyme, lactoferrin, histamine, and leukocytes [10,11,12]. The occurring changes in the level of steroid hormones also reduces the intestinal absorption of calcium into the body, which in turn leads to disturbances in the regulation of calciumphosphate metabolism, resulting in increased release of calcium into both blood serum and saliva [5,13,14]. A significantly higher concentration of calcium in the saliva of menopausal women may therefore lead to a faster mineralization of plaque and, consequently, increased calculus formation, which has an undoubted effect on the progression of gingivitis and periodontitis [15,16,17].

The aim of this study was to estimate differences in selected physicochemical properties of saliva between menopausal and premenopausal women.

## 2. Materials and Methods

### 2.1. Patients’ Population

The study population consisted of 9 menopausal women, aged 48–55 years, previously gynecological diagnosed in the Gynecological and Obstetric Ambulatory Clinic, Family Medicine Center of the Medical University of Gdansk, Poland, who volunteered for a follow-up examination of periodontium and oral mucosa at the Department of Periodontology and Oral Mucosa Diseases, and 15premenopausal women, aged 20–30 years, as the control group. All groups included generally healthy, non-smoker women. Patients with diseases that might interfere with the conditions of the oral mucosa, like diabetes, disorders of salivary secretion, women taking Hormone Replacement Therapy or any medications permanently and treated with antibiotics or steroid preparations in the last 6 months were excluded from the research.

The study protocol was approved by Ethics Committee of the Medical University of Gdansk, Poland (NKBBN/341/2019). Ethical aspects of the research followed the World Medical Association Declaration of Helsinki.

### 2.2. Saliva Collection

Mixed unstimulated whole saliva was collected from each patient studied. The saliva of premenopausal women was collected in the preovulatory part of the menstrual cycle after the end of menstruation. All samples were collected into a sterile silicone Corning-type test tube in the morning hours, two hours after the last intake of food or drinks. Unstimulated salivary samples were obtained by expectoration in the absence of chewing movements.

The samples were clarified by centrifugation (2000× *g*; 10 min) and immediately stored at −20 °C for the later determination of immunoglobulin A, lactoferrin, lysozyme, and ionized calcium.

To calculate the rate of resting saliva, the amount of saliva collected over 6 min was divided by 6 to give the amount of saliva secreted per minute (mL/min).

The following scale of values was adopted:

1. 0.26–0.30 mL/min—proper unstimulated salivation
2. 0.25–0.1 mL/min—reduced unstimulated salivation (oligosialy)
3. <0.1 mL/min—impaired salivation–kserostomy

The concentration of hydrogen ions was determined in unstimulated saliva immediately after its collection using a Fisher pH-meter with a glass electrode.

### 2.3. Analysis of Saliva 

The whole mixed unstimulated saliva collected from menopausal women and women in the control group was analyzed in the biochemical laboratory of the Conservative Dentistry Department, Medical University of Gdansk, Poland. The concentration of immunoglobulin A, lactoferrin, and lysozyme were analyzed in centrifuged (10,000× *g*, 10 min) unstimulated saliva by ELISA technology, using commercially available kits (Sigma–Adrich, St. Louis, MO, USA).The level of ionized calcium was determined by the Arsenazzo III method, which involves the use of the Arsenazzo III metallochromogen, which, by binding calcium ions, produces a colored complex whose absorbance can be measured at 650 nm. Metallochromogen Arsenazo III has a strong affinity for calcium ions and does not interfere with other cations normally found in serum, plasma, urine, or saliva. The color intensity measured at 650 nm is proportional to the calcium concentration in the sample tested. A reagent from Alpha Diagnostics (pH = 6.75) was used for the experiment, containing the active ingredients Arsenazo III (0.2 mmol/L) and imidazole buffer (100 mmol/L). The Alpha Diagnostics reagent containing calcium at 2.5 mmol/L, ready for direct use, was also used for the standard curve [18].

The reagent was added to the saliva sample in a ratio of 1:50, and the mixture was incubated for 1 min. The absorbance was then measured at 650 nm. Calcium concentration was calculated from the standard curve [18].

### 2.4. Statistical Analysis 

The statistical analyses were performed using the statistical suite STATISTICA (data analysis software system), version 12.0 (StatSoft. Inc., Tulsa, OK, USA).

Statistical significance of the differences between the two groups wereprocessed with the U Mann–Whitney test. In all calculations, the statistical significance level of *p* < 0.05 was used.

## 3. Results

Table 1 presents the value of pH, salivary flow rate, and concentrations of lactoferrin, lysozyme, immunoglobulin A, and ionized calcium for unstimulated saliva from menopausal women and the control group.

The value of salivary flow rate in menopausal women was 0.3 ± 0.2 mL/min (range 0.1–0.5, median (Me) = 0.3) and that in the control group was 0.8 ± 0.3 mL/min (range 0.3–1.5, Me = 0.8). Statistical analysis revealed a significant difference between menopausal and premenopausal women to the disadvantage of menopausal women.

Statistically significant differences were also observed between study group and premenopausal women for the concentration of lysozyme in the saliva. The concentration of lysozyme in menopausal women was 2.6 ± 2.3 µg/mL (range 0.5–8.3, Me = 2.1) and in the control group was 6.5 ± 4.8 µg/mL (range 1.3–22.1, Me = 4.8). The concentration of lysozyme in the saliva of premenopausal women was distinctly higher than that in the saliva of women in the study group.

**Figure 1:**
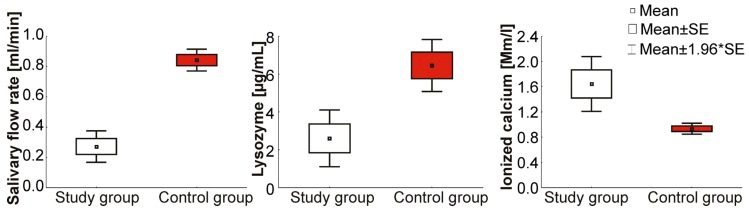
Mean values of unstimulated salivary flow rate and concentrations of lysozyme and ionized calcium in menopausal women and the control group. Legend: SE-Standard Error, *-statistically significant values.

In relation to ionized calcium concentration, statistically a significant difference wasfound between premenopausal and menopausal women to the advantage of the latter. The concentration of ionized calcium in the study group was 1.6 ± 0.7 Mm/L (range 1.1–3.3, Me = 1.5) and thatin the control group was 0.9 ± 0.3Mm/l (range 0.5–1.9, Me = 0.9). The concentration of ionized calcium in the saliva of menopausal women was distinctly higher than that in the saliva of women in the control group (Figure 1).

## 4. Discussion

Saliva secretion is the principal defense factor in the oral cavity. Consequently, a low salivary flow has detrimental effects on teeth and the oral mucosa. A normal flow of unstimulated and stimulated saliva is important to ensure sufficient and continuous lubrication of the oral tissues. Moreover, the fluid characteristics of saliva are essentials for dissolving taste substances and transporting them to taste receptor sites, food bolus formation, facilitation of mastication, swallowing, as well as speech. The moist environment is also important for the colonization and growth of microorganisms on oral surfaces [10,11,12]. Insufficient saliva can result in the disruption of the microbial balance to the benefit of certain pathogens such as *Candida albicans* and *Streptococcus mutans* [11]. Higher occurrence of dental caries, oral mucositis, dysphagia, oral infections, and altered taste has been reportedin those with reduced salivary flow [19].

Oral dryness is one of the most common oral symptoms reported by menopausal women. The prevalence and severity of symptoms may not be proportional to the amount of saliva secreted; however, multiple studies indicate the presence of estrogen receptors in the salivary glands, whose activity can possibly be affected in menopause due to decreased level of estrogen during this time [2,5,6,7,8]. In our studies of menopausal women, a decrease in salivary flow rate in comparison to premenopausal women was observed. Although the result obtained for menopausal women was within the norm of salivation rate, it was closer to its lower limit, and its value was almost three times lower than that obtained for the control group. Research conducted by Maheshetet. et al. [19] and Foglio-Bondaet et al. [20] obtained similar results, indicating that salivary flow rate was also significantly lower in menopausal women in comparison with menstruating women. Also, studies provided by Rukmini [21] showed a noticeable diminution in the salivary flow rate and pH of saliva in menopausal women, noting the correlation between the reduction of these parameters and increased bacterial plaque deposition and, therefore, risk of periodontitis. However previous studies on the effect of menopause on salivary flow rate have reported diverse results. Kullanderet et al. [22] also reported lower secretion rates in menopausal women than in menstruating women, while studies by Ship et al. [23] and Ben Aryeh [1] did not find any significant changes.

Besides its physicochemical properties, saliva also plays an important role in the immune defense of the oral cavity. Lysozyme is part of the innate salivary defense mechanisms. It is a small (145 kDa) protein present in body fluids, including saliva. Salivary lysozyme is produced by the salivary glands and also by neutrophil granulocytes entering the mouth. It is an enzyme that causes the lysis of bacterial cell walls and is also present in the gingival crevicular fluid. In addition to its antibacterial activity, it also presents antiviral and antifungal activity [10,11,12,24,25,26].

In our research, statistically significant differences were observed between menopausal and premenopausal women as concern the concentration of lysozyme in the saliva. The concentration of lysozyme in the saliva of menopausal women was distinctly lower than that in the saliva of the control group. According to Rukmini [21], long-lasting instabilities such as aging as well as autoimmune diseases and medical treatment might be estimated to change salivary protein concentrations, but it has been found that the concentrations of salivary antimicrobial elements do not drop with age in unmedicated patients, as shown by Rudney [27]. No other studies related to statistically significant changes in salivary lysozyme concentration in menopausal women have been found. However, research provided by Leimola-Virtanen et al. [7], although based on smallpatient numbers, indicate that the protein composition of saliva in post- and perimenopausal women is estrogen-dependent. Further wider researchin this field should be carried out. Decreased lysozyme concentrations in the saliva have also been observed in patients with periodontitis (Ito and et al. [28]), patients suffering from decompensated type 2diabetes (Chorzewski et al. [29]), and smokeless tobacco users (Rudney [27]). Due to the antibacterial and antiviral properties of salivary lysozyme and also to it stability to inhibit the growth of *Candida* (Lal et al. [30]), its decreased level in the saliva of menopausal women may affect the increased risk of oral candidiasis, caries, periodontitis, and other oral infections in this significant group of female patients. Further wider research should be conducted on this topic.

The saliva concentration of ionized calcium presented another statistically significant difference between the postmenopausal women seen in our current study andthe control group. Decreased concentration of estrogens, which occurs in the menopausal period, reduces intestinal calcium absorption, which leads to an increase in serum parathyroid hormone levels (PTH). PTH is a hormone that is responsible for the regulation of calcium and phosphate metabolism in the body. During the menopausal period, the increased release of calcium into serum is reflected in the increased concentration of calcium in saliva [5,13,14]. In our research, the concentration of ionized calcium in the saliva of menopausal women was distinctly higher than that in the saliva of women in the control group. Agha-Hosseini et al. [14] also confirmed the higher concentration of calcium in the saliva of menopausal women in their studies. Sewón et al. [13] as well showed similar results. A study from Iran on salivary flow rate and composition of 42 menopausal women with or without xerostomia (21 cases, 21 controls) showed that the mean calcium concentration was significantly higher in the cases than in the controls. In relation to the increased level of ionized calcium in the saliva, several studies have shown that subjects with increased inorganic salivary calcium, pH, flow rate, and poor oral hygiene are at a higher risk of developing periodontitis, as plaque calcification occurs more readily in such patients [15,16,31,32]. Furthermore, these individuals are more resistant to caries and have an increased number of intact teeth due to the increased remineralization potential of their saliva. Salivary calcium increases the level of plaque calcium and is therefore readily available for remineralization, causing a low caries incidence. (Rajesh et al., Fiyaz et al. Sewón et al. [15,16,33]). In the light of these results, menopausal women in the presence of the describe dconditions in the oral cavity may be at higher risk of developing periodontitis but may have more intact teeth.

The limitations of our study definitely include the very small sample of menopausal women examined. Statistically significant differences in the results were already obtained with such a small group of patients, which clearly encourages further research, widening the sample and possibly further assessing the clinical picture of the mucosa and periodontal tissues, as well as studying differences in the oral microbiota.

## 5. Conclusions

The saliva of menopausal women appeared significantly different from that of the control group. Differences in physicochemical parameters such as the salivary flow rate and concentrations of lysozyme and ionized calcium were observed. These differences in saliva properties observed in menopause can potentially affect the oral environment of women in this particular period, possibly increasing the risk of some pathological changes in the oral cavity and consequently indicating the need to take special care of this group of female patients in order to help them maintain a proper oral health. Dentists and gynecologists should be aware of the problems associated with menopause and need to provide these women complete health care, including dental care as an integral part.

## Figures and Tables

**Table 1:** Values of pH, salivary flow rate, and concentrations of lactoferrin, lysozyme, immunoglobulin A, and ionized calcium for unstimulated saliva from menopausal women and the control group.

	Study Group (*N* = 9)	Control Group (*N* = 15)	*p*-Value
pH			0.3843 ^1^
Mean ± SD	6.5 ± 1.1	7.5 ± 8.0	
Range	4.5–8.0	5.0–61.0	
Me	7.0	6.3	
Salivary flow rate (mL/min)			0.0001 ^1,^*
Mean ± SD	0.3 ± 0.2	0.8 ± 0.3	
Range	0.1–0.5	0.3–1.5	
Me	0.3	0.8	
Lactoferrin (µg/mL)			0.6717 ^1^
Mean ± SD	6.1 ± 5.3	7.0 ± 8.8	
Range	1.2–17.3	1.1–61.7	
Me	4.0	5.6	
Lysozyme (µg/mL)			0.0019 ^1,^*
Mean ± SD	2.6 ± 2.3	6.5 ± 4.8	
Range	0.5–8.3	1.3–22.1	
Me	2.1	4.8	
Immunoglobulin A (µg/mL)			0.2944 ^1^
Mean ± SD	416.4 ±389.0	515.8 ± 429.6	
Range	71.0–1 245.0	36.0–2 182.0	
Me	251.0	399.0	
Ionized calcium (Mm/L)			0.000 ^1,^*
Mean ± SD	1.6 ±0.7	0.9 ± 0.3	
Range	1.1–3.3	0.5–1.9	
Me	1.5	0.9	

Legend: ^1^ test U Mann–Whitney, * *p* < 0.05, mean values, SD-standard deviation, Me-median.
